# Supplementary material for: AI adoption in E-commerce enterprises: Insights into current practices and future directions from an interview study
Source: PLoS One. 2026 Mar 12;21(3):e0336416. doi: 10.1371/journal.pone.0336416 (PMC12981462; doi:10.1371/journal.pone.0336416)
Supplement: S1 File — (DOCX) [file pone.0336416.s001.docx]

**Interview Questions**

The purpose of this interview is to understand your company's current application of artificial intelligence technology in e-commerce, as well as your views and suggestions on this topic. This information will provide valuable data support for our research, and we hope that through your participation, we can better explore how to leverage artificial intelligence technology to enhance the e-commerce efficiency and effectiveness of small and medium-sized enterprises. The interview will last approximately 30-45 minutes. We will discuss several key questions, and you are free to answer based on your actual situation. All interview content will be kept strictly confidential and used only for academic research; your personal and company information will not be disclosed. Before we start the interview, I would like to learn more about your background. Please take three minutes to introduce your role in the company, including your years of experience and job responsibilities.

Time check: 5 minutes.

Q1: Current Application Status: What types of AI technologies are currently being used in your enterprise’s e-commerce operations, and how are they integrated into business processes and platforms?

1) What is your enterprise’s primary e-commerce platform? Why did you choose this platform?

2) You mentioned the use of (technology name). In which specific processes of the

enterprise is it applied (for example: advertising placement, data analysis)?

3) What channels did your enterprise acquire or access these technologies? (for example: internal development, external procurement, or third-party collaboration)

4) Have you used any free tools or AI platforms? If so, which ones specifically?

Time check: 10 minutes.

Q2: Application Effectiveness: How effective have these AI applications been in supporting or improving your e-commerce operations?

1) In what areas have you observed the most significant benefits from AI usage? (for example: cost reduction, efficiency, accuracy)

2) You mentioned that the effects are significant/average; in what specific aspects is this reflected?

3) What specific cases or data can illustrate the effectiveness of these technologies?

4) Have there been any discrepancies between your expectations and the actual results of using AI technologies? If so, what were the causes or underlying challenges?

Time check: 10 minutes.

Q3: Influencing Factors: What factors affect a company's decision to choose or not choose AI technology in e-commerce? How do the current market environment or policy support impact a company's adoption of AI technology, providing opportunities or limitations?

1) Do companies believe that the implementation costs of AI technology (initial investment, maintenance costs) have a direct impact on their decision-making?

2) Does the company have an internal technical team or professional capabilities to support the implementation of AI technology?

3) Does the current industry competitive environment encourage companies to adopt AI technology more quickly? Are competitors already using AI?

4) Are there government or industry association policies or incentives that encourage companies to adopt AI technology, such as tax breaks or funding subsidies?

5) If you were to list the three most important influencing factors, how would you rank them? Why?

Time check: 10 minutes.

Q4: Future Plans and Experience Sharing: In the future, does your company intend to further increase its investment in AI technology? What are your expectations for the continuous innovation of artificial intelligence technology, especially in the area of e-commerce? Does your company have any successful cases of applying AI technology in e-commerce that you can share?

Time check: 5 minutes.

Thank you very much for taking the time to share your valuable experiences and insights with us; this provides important support for our research. If there are any areas that need further clarification or confirmation in the subsequent research, I may reach out to you again. You are also welcome to contact us anytime to provide new ideas or feedback. Thank you again for your help.
