# Supplementary material for: AI adoption in E-commerce enterprises: Insights into current practices and future directions from an interview study
Source: PLoS One. 2026 Mar 12;21(3):e0336416. doi: 10.1371/journal.pone.0336416 (PMC12981462; doi:10.1371/journal.pone.0336416)
Supplement: Attachment — Submitted filename: renamed_c1794.doc [file pone.0336416.s002.doc]

This study employs the TOE and TAM theoretical frameworks to explore the current application status, effectiveness, and challenges of AI technology through interviews with multiple e-commerce enterprises in Anhui Province. The research design is reasonable, the data is solid, and the findings offer certain insights. However, improvements are needed in theoretical depth, methodological rigor, and clarity of exposition.

1.Introduction Section:①The description of the research gap is somewhat broad and lacks systematic understanding. It does not precisely align with the design of this study.②The logical connection between the proposed multidimensional interaction of "technology-organization-environment-perception" and the integration of the TOE and TAM frameworks is unclear.

2.Literature Review:①It fails to review literature related to "stratified AI adoption," resulting in a lack of academic dialogue foundation for a core finding.②While it cites the TOE and TAM frameworks extensively, it lacks a critical discussion on how these two frameworks are combined at the organizational level. It is recommended to supplement the rationale for selecting these two frameworks, their complementarity, and their potential limitations in studying organizational AI adoption.

3.Methodology:①Sample information is incomplete. Details such as the industry type, revenue scale, and platform operation duration of the enterprises are missing.②The rationale for selecting Anhui Province is not provided. Can the level of e-commerce development in Anhui Province represent the general situation nationwide? A brief background explanation is needed to enhance the external validity of the sample.

4.Interview data findings & analysis:①A serious contradiction exists between Figure 2, where "Content Generation" accounts for only 6%, and the textual description in the results section stating that it is "widely and commonly used" by enterprises.

5.Discussion & Comparative Analysis:①The fifth key finding (dynamic adaptation mechanism) lacks a corresponding thematic section in the results, making its introduction in the discussion seem abrupt and poorly connected.②The comparison with literature lacks specific references. For instance, the claim that "existing literature assumes AI is a standardized solution" is not supported by citations to specific studies.

6.Conclusion:①The research limitations are not explicitly stated. It is recommended to supplement future research directions based on the gaps identified in this study.

7.Formatting and Details:Inconsistent formatting is present in the references section. Please strictly adhere to the journal's required style.
