# Supplementary material for: AI adoption in E-commerce enterprises: Insights into current practices and future directions from an interview study
Source: PLoS One. 2026 Mar 12;21(3):e0336416. doi: 10.1371/journal.pone.0336416 (PMC12981462; doi:10.1371/journal.pone.0336416)
Supplement: Attachment — Submitted filename: Response to Reviewers.docx [file pone.0336416.s003.docx]

Dear Editor and Reviewers,

Thank you very much for your time and effort in reviewing our manuscript titled " AI Adoption in E-commerce Enterprises: Insights into Current Practices and Future Directions from an Interview Study ". We truly appreciate the constructive comments and insightful suggestions provided by the reviewers, which have been immensely helpful in improving the quality and clarity of our paper.

We have carefully considered each of the points raised and have revised the manuscript accordingly. In the following sections, we provide a point-by-point response to the reviewers' comments. The changes in the revised manuscript have been highlighted using Red Color for your convenience.

**Response to academic editor**

1. The manuscript has been formatted in accordance with the requirements. However, the paragraph spacing has not been changed to double spacing for the sake of easier reading.

2. In this study, informed consent was obtained verbally from all participants prior to each interview. Before interview began, the interviewer explained the study purpose, voluntary nature of participation, confidentiality, and anonymization procedures using a standardized introductory script (see article Appendix). Participants were explicitly asked whether they agreed to participate and to be audio-recorded for academic research purposes. Participants’ agreement was captured on the audio recording at the beginning of each interview.

According to institutional guidelines, this study—based on professional interviews with enterprise representatives and involving no personal, medical, or vulnerable population data—was classified as minimal-risk social science research and did not require formal Institutional Review Board (IRB) approval. This exemption applies to studies collecting non-sensitive professional opinions without personal identifiers. We have clarified this procedure in the Methodology section accordingly.

3. We have revised the Funding Statement in accordance with PLOS ONE’s requirements. The amended Funding Statement is provided below for your update of the submission system:

Funding Statement:

This research was supported by the project “Application Research of Artificial Intelligence Technology in E-Commerce Enterprises in Anhui Province” (Fund Number: 2024AH052502). There was no additional external funding received for this study. The funder had no role in the study design, data collection and analysis, decision to publish, or preparation of the manuscript.

We confirm that no other institutional, commercial, or personal financial support was received during the conduct of this study.

4. We have removed all funding-related text from the Acknowledgments and all other sections of the manuscript, in accordance with PLOS ONE’s policy.

5. We confirm that our submission now includes the minimal data set required to replicate the findings of this qualitative study. We have deposited an anonymized dataset on Zenodo (DOI:[10.5281/zenodo.18298170](https://doi.org/10.5281/zenodo.18298170)) containing a structured Excel file that summarizes all interview-derived evidence underlying the reported themes, figures, and conclusions. This dataset includes: The coded thematic summaries for each of the eight enterprises; The values used to construct all figures (such as, frequency of AI application domains, main platform); Metadata describing the operation and application status of the enterprise.

Full verbatim transcripts are not publicly shared due to ethical considerations and participant confidentiality. However, the deposited dataset represents the minimal de-identified data necessary to reproduce all analytical steps and results reported in the manuscript.

6. We have now finalized a data-sharing plan in accordance with PLOS ONE’s open data policy. All data underlying the findings have been deposited in a stable public repository (Zenodo) and are freely accessible at: (DOI:[10.5281/zenodo.18298170](https://doi.org/10.5281/zenodo.18298170)). The dataset is fully anonymized and does not contain personal identifiers or sensitive information.

7. We have revised the manuscript to ensure that the ethics statement appears only in the Methodology section. All ethics-related text has been removed from any other sections of the manuscript.

**Response to reviewer #1**

1. We substantially strengthened the transparency of how interview data were transformed into themes and higher-level conclusions. First, we expanded the Methodology section to specify each step of the qualitative procedure, detailing how open codes were generated from raw transcripts, how they were aggregated into axial categories, and how these categories were mapped onto higher-order themes using TOE and TAM as sensitizing concepts. Second, in the Results section, we revised each thematic subsection to directly link major claims to their underlying codes and representative quotations from multiple enterprises. Third, we added summary tables that present enterprise profiles and the correspondence between codes, themes, and interview evidence. These tables provide a transparent basis for tracing how conclusions were grounded in the data.

2. In the revised manuscript, we substantially expanded the Methodology section to provide a transparent account of our qualitative procedures. We now specify that the thematic analysis followed a hybrid strategy, combining deductive and inductive coding. The TOE and TAM frameworks are explicitly described as sensitizing concepts guiding initial coding, while allowing new themes to emerge from the data. We further clarify how codes were developed, refined, and grouped into themes, and how these themes were linked to the analytical framework. To enhance trustworthiness, we detail the use of member checking, cross-review of coding by a second researcher. These additions enable readers to better evaluate the rigor, transparency, and robustness of the analytic process.

3. We have clarified the Data Availability Statement and now provide a de-identified dataset summarizing the interview materials as Supporting Information and in a public repository. This dataset constitutes the minimal data set required to reproduce all reported findings.

4. We have moved and expanded the ethics statement within the Methodology section. The revised text now explicitly describes the ethical pathway, including the use of verbal informed consent, how consent was documented (audio-recorded at the beginning of each interview), the anonymization procedures, data storage, and the basis for exemption from formal IRB review under institutional guidelines for professional, non-sensitive interviews. This ensures transparency and compliance with journal requirements.

5. We carefully edited the entire manuscript to improve clarity, precision, and readability in English. In addition, we added explicit statements in the ‘Sample Selection and Research Context’ section to clarify the limited sample size and the single-province setting, thereby tempering any unintended claims of broad representativeness. We also revised Figure 2 and its accompanying text to ensure that all quantitative-looking results are clearly presented as descriptive summaries of interview data.

**Response to reviewer #2**

1. Introduction Section

We have revised the Introduction to moderate the scope of the research gap and to ensure closer alignment with the study design. The revised version highlights the limited empirical insight into how AI applications are operationalized across platforms, processes, and enterprise contexts in e-commerce. The revised Introduction explains their complementary roles: TOE provides a structural perspective for examining technological, organizational, and environmental conditions, while TAM contributes a perceptual perspective for understanding how enterprises evaluate the effectiveness of AI applications in practice. This clarification supports the proposed multidimensional interaction of “technology–organization–environment–perception” and ensures a clearer alignment between the theoretical framework and the research design.

2. Literature Review

First, to respond to the absence of prior work on “stratified AI adoption,” we expanded Section ‘AI Adoption in E-commerce’ to incorporate multidisciplinary reviews, industry surveys, and SME-focused studies that document uneven and layered patterns of AI adoption across firms. The revised text now synthesizes evidence showing that AI diffusion and its benefits are distributed asymmetrically, shaped by firm size, digital readiness, and resource endowments.

Second, in response to the concern regarding the conceptual integration of TOE and TAM, we substantially revised Section ‘TOE & TAM in AI Adoption’. The revised section now explicitly clarifies:

(1) The rationale for selecting TOE and TAM—TOE captures structural and contextual conditions, while TAM explains evaluative perceptions shaping adoption;

(2) Their complementarity at the organizational level, where perceived usefulness and ease of use are interpreted as collective managerial evaluations rather than individual attitudes;

(3) Their analytical boundaries in organizational AI adoption research. We now explicitly acknowledge that TOE emphasizes contextual structures without explicating internal evaluation mechanisms, whereas TAM focuses on evaluative perceptions without modeling organizational resources or environmental constraints. The combined use of TOE and TAM is therefore positioned as an analytically balanced approach that links contextual conditions with organizational-level perceptions, while situating the analysis at the level of enterprise adoption decisions rather than broader inter-organizational dynamics or long-term capability evolution.

3. Methodology

We have revised the Methodology section to provide more complete and transparent sample information. A new table (Table 1) has been added to present the basic profiles of all interviewed enterprises, including business type, industry background, organizational scale, and key operational characteristics. This addition clarifies the heterogeneity of the sample and strengthens the basis for cross-case comparison.

In addition, we have expanded the description of the regional context to justify the selection of Anhui Province. The revised manuscript now explains that Anhui represents a transitional e-commerce environment with development indicators close to the national average, combining both emerging and mature digital practices. This positioning enhances the external validity of the study by situating the cases within a region that reflects common conditions faced by many Chinese e-commerce enterprises, rather than an extreme or atypical context.

4. Interview data findings & analysis

We have revised the figure by replacing the original pie chart with a bar chart to more accurately reflect the nature of the data. Because individual enterprises often apply AI across multiple functional areas, the values represent the number of enterprises that mentioned each application domain rather than proportional shares.

We have also added explicit textual clarification in Section ‘AI Applications in E-commerce Processes’ to explain the meaning of the figure and its statistical logic, emphasizing that the frequencies indicate the relative prevalence of AI use across domains.

5. Discussion & Comparative Analysis

(1) To address this issue, we have revised the structure of the Interview Data Findings & Analysis chapter by adding a new subsection ‘Application Pathways and Modes of AI Use’. This subsection is positioned before the analysis of application effectiveness and is explicitly grounded in interview evidence. It systematically reports how enterprises of different sizes integrate AI into their e-commerce operations Based on these findings, the fifth key point in the Discussion “The deployment of AI technology demonstrates a dynamic adaptation mechanism and a paradigm of human–machine collaboration” has been revised and now directly builds upon Section ‘Application Pathways and Modes of AI Use’. The discussion no longer introduces this concept in isolation but synthesizes patterns already identified in the Results. In addition, representative interview excerpts illustrating platform dependence, tool augmentation, and in-house development have been added to the interview summary table to strengthen the audit trail between data, themes, and conclusions.

(2) Based on your suggestions, we have added the missing references in Comparative Analysis: Interview Findings vs. Literature Review, and marked them.

6. Conclusion

We have revised the Conclusion section to explicitly articulate the limitations of the study, including the restricted sample scope, the qualitative and self-reported nature of the data, and the cross-sectional design. In addition, we have added a dedicated paragraph outlining concrete directions for future research, such as multi-regional expansion, mixed-methods validation, and longitudinal investigation of AI adoption.

7. Formatting and Details

By comparing with the formatting requirements, the references and other formats in the article were adjusted accordingly.

Once again, we would like to express our sincere gratitude to the reviewers for their invaluable feedback. We believe the manuscript has been significantly strengthened through this revision process. We hope that the revised version meets the high standards of PLOS ONE. Please do not hesitate to contact us if any further clarification or additional modification is required. We look forward to hearing from you.

Sincerely,

Tong Zhu, Mohd Zaidi Abd Rozan
