# Supplementary material for: AI adoption in E-commerce enterprises: Insights into current practices and future directions from an interview study
Source: PLoS One. 2026 Mar 12;21(3):e0336416. doi: 10.1371/journal.pone.0336416 (PMC12981462; doi:10.1371/journal.pone.0336416)
Supplement: Attachment — Submitted filename: Response_to_Reviewers_auresp_2.docx [file pone.0336416.s004.docx]

Dear Editor and Reviewers,

Thank you for your message and for the opportunity to submit the revised version of our manuscript. In this revision, we have carefully reviewed the manuscript in accordance with the journal’s requirements. Specifically:

1. We conducted a thorough check of the entire reference list to ensure completeness, formatting consistency, and accuracy.
2. We identified that one previously cited article (Reference 5) had been retracted. This reference has been removed and replaced with relevant reference that is recent in content.
3. We verified and supplemented DOI information where necessary to ensure that all references are properly documented.

We appreciate the constructive review process and thank you and the reviewers for your guidance. We look forward to your final decision.

Sincerely,

Tong Zhu, Mohd Zaidi Abd Rozan
